# Paeoniflorin ameliorates chronic stress-induced depression-like behavior in mice model by affecting ERK1/2 pathway

**DOI:** 10.1080/21655979.2021.2003676

**Published:** 2021-12-07

**Authors:** Meiling Tang, Min Chen, Qiang Li

**Affiliations:** a Department of Nursing, Qiqihar Medical University, Qiqihar, Heilongjiang, China; b Department of Enrolment and Employment, Qiqihar Medical University, Qiqihar, Heilongjiang, China

**Keywords:** Paeoniflorin, chronic restraint stress, depression, in vivo, ERK1/2 pathway, treatment

## Abstract

Depression is a mental and emotional disorder that has made an opening great burden to the society. Paeoniflorin showed remarkable antidepressant-like effects in multiple animal models with depressive disorders. However, the molecule of paeoniflorin on depression is less studied. This study aims to explore the effect and the molecular mechanism of paeoniflorin on depression in a chronic restraint stress (CRS) mice model. CRS model of C57BL/6 J mice was set up. Sucrose preference test (SPT), tail suspension test (TST), open field test (OFT) and forced swimming test (FST) were used to assess depression symptoms. Immunofluorescence staining, quantitative reverse transcription-polymerase chain reaction (qRT-PCR) and western blotting were implemented to detect the expression changes of the proteins involved in extracellular signal-regulated kinase 1/2 (ERK1/2) signaling pathway. Results showed that paeoniflorin treatment decreased the degree of depression in the CRS mice. Further analysis showed that the expression of ERK1/2 proteins was significantly downregulated, while paeoniflorin could elevate the expression of ERK1/2 proteins in CRS mice. Finally, it showed that inhibiting signaling ERK1/2 pathway could aggravate the depressive behavior when treatment with ERK-specific inhibitor U0126, while the condition could be partially relieved when treated with paeoniflorin. In conclusion, the present study demonstrated that paeoniflorin attenuated chronic stress-induced depression-like behavior in mice by affecting the ERK1/2 pathway. These findings provided the basis for the molecular mechanism of paeoniflorin on the effect of depression, which support paeoniflorin might act as an important drug in the treatment of depression.

## Introduction

With the development of social stressors, the incidence of depression is also increasing year by year in the world caused by increasing pressure of people’s life [1]. According to the latest epidemiological survey, the incidence of depression has accounted for 1.4% of the world, and 4 million people commit suicide every year because of depression [2,3]. It has caused a great burden to the collective economy [4]. Depression is a mental and emotional disorder, and its main clinical manifestations are low mood, anhedonia, decreased social behavior, and increased negativity [5]. The pathogenesis of depression is quite complicated, and the neurobiological pathogenesis of depression is still unclear. The current classic pathogenesis of depression is the insufficient function of the serotonergic system and monoaminergic nervous system [6]. However, the treatment of depression based on this hypothesis does not present effective treatments, and exploring the molecular mechanism of depression is an inevitable way to solve the current clinical problems.

Paeoniflorin is the main effective component of the traditional Chinese medicine Paeonia lactiflora and tree peony. A large number of studies have proved that paeoniflorin has anti-inflammatory, analgesic, antitumor, liver protection, nerve protection, immune regulation, sedative and hypnotic effects [7–11]. Paeoniflorin was proved to ameliorate atherosclerosis by suppressing TLR4-mediated NF-κB activation [12]. Ji et al. demonstrated that paeoniflorin suppressed TGF-β mediated epithelial-mesenchymal transition in pulmonary fibrosis by upregulating the expression of Smad7 [13]. In addition, several signaling pathways regulated by paeoniflorin were also investigated, such as LKB1/AMPK and AKT pathways in fructose-induced insulin resistance and hepatic steatosis [14], Bax/Bcl-2/caspase-3 signaling pathway in chondrocyte apoptosis [15] and RhoA/ROCK signaling in glioblastoma [16]. Extracellular signal-regulated kinases (ERKs) are an important type of mitogen-activated protein kinases (MAPKs) that have been proved play a pivotal role in regulation of depression and hippocampus neurons [17]. However, whether ERK signaling pathway affected by paeoniflorin remains unclear.

With continuous extensive research on paeoniflorin, increasing numbers of studies have found that paeoniflorin plays an important role in depressive-like behavior. Such as total glycosides of peony alleviated depression induced by chronic unpredictable stress by inhibition of monoamine oxidases and the attenuation of oxidative stress in mouse brain [18]. Liu et al. investigated the effects of paeoniflorin administration on depression-like behaviors and cognitive abilities in mice subjected to chronic unpredictable mild stress (CUMS) and found that paeoniflorin attenuated impairment of spatial learning and hippocampus long-term potentiation in mice subjected to CUMS [19]. Recently, Li demonstrated that paeoniflorin ameliorated depressive-like behavior in prenatally stressed offspring by restoring the HPA axis- and glucocorticoid receptor-associated dysfunction [20]. However, the regulation mechanism of paeoniflorin in depressive-like behavior remains unclear. The effects of paeoniflorin on depression and the ERK signaling pathway have not been studied.

Therefore, the purpose of the present study was to explore the effects of paeoniflorin on the CRS-induced depression-like behavior in mice model and investigate the mechanisms by which novel signaling pathways are regulated. These findings provide the basis for the molecular mechanism of paeoniflorin on the effect of depression, which support paeoniflorin might act as an important drug in the treatment of depression.

## Materials and methods

### Animals and CRS model

Male C57BL/6 J mice weighted at 19–23 g at 6–9 weeks were recruited from Charles River Laboratories (Beijing). All the mice were fed in the SPF environment at 20–24°C with 12 h light/12 h dark under 35–50% humidity. The CRS model was established according to the previous report [21]. Briefly, mice were acclimated to 1% sucrose for 2 days before modeling. The mice were randomly divided into two groups (*n* = 8) as control group and CRS group according to the SPT. The mice in the CRS group were restrained in a perforated transparent syringe and were restrained for 3–4 h every day and last for 10 days. During the process of CRS, all mice are free of trauma and pain and restrained mice were maintained in a quiet, dark environment. The mice were allocated to their cages with free access to water and food after the restraint immediately. Depression degree of mice was evaluated by FST, SPT and TST. For the functional analysis of paeoniflorin to chronic stress-induced depression-like behavior, the mice were intraperitoneal injected i.p. 1 h with 10, 30, 60 and 120 mg/kg paeoniflorin once daily before the test for 5 consecutive weeks. In the last week of CRS, 1 µL/min U0126 was injected in the lateral ventricle once per day for 5 min as described in existing reference with minor modification [22]. Control mice received no stimulus. Behavioral assessment was performed between 11:00 and 15:00 after week 4, when the administrations of paeoniflorin were ceased. Each testing session was arranged in the same order, with the researchers blinded to the experimental conditions.

### Sucrose preference test (SPT)

SPT had to be carried out according to the previous report (Crocetin ameliorates chronic restraint stress-induced depression-like) [23]. Briefly, mice were acclimated to sucrose to eliminate the position preference of sucrose before the experiment. Firstly, a tap water pipe with the capacity of 50 ml is placed on one side of a breeding cage and a 50 ml sucrose tube are placed on the other side. After weighting the tap water pipe and the 1% sucrose water pipes, place the sugar pipe and water pipe on the left and right sides of the breeding cage randomly. The mice were allowed to drink freely for 12 h and, the weight of the water pipe begins to be formed again after 12 h. The consumption of tap water and sugar water in the 12 h of the test period was calculated and the sucrose preference rate was calculated. Sucrose preference rate = sucrose water consumption/total consumption of sucrose water and tap water 100%. The sucrose preference rate was compared with the normal group.

### Tail suspension test (TST)

As previously described [24], TST was performing at 8:00 am the next day after the SPT. The adhesive tape is stuck to the tail end of the mouse about 1 cm and is hung on a plastic rod and the distance from the ground to the head is controlled to be about 50 cm in length. The mice were filmed with a high-definition video camera for 6 minutes and then the video was replayed on a computer and the activity time of the mice after 4 minutes was counted by manual objective blind method. The mice did not move their limbs at all or moved very slightly to act as quiescence. During the test, mice were kept separated from each other to prevent possible visual and acoustic associations.

### Open field test (OFT)

General locomotor activity was measured using OFT according to the methods previously described [25]. Briefly, the OFT apparatus which consisted of a 60×60×40 cm square boxes was divided into 25 equal-size squares. Each mouse was gently placed in a corner of the apparatus, observed for 5 min, and a count was taken into account in mouse crossed from one square to the next. The number of fecal was also recorded during the 5 min by ANY-maze system (Stoeling, USA). After each trial, the apparatus was cleaned using 70% ethanol.

### Forced swimming test (FST)

The FST was carried out according to the previous report [26]. The C57BL/6 J mice were placed into a transparent glass tube (35 cm high, 15 cm diameters), with a water depth of 10 cm and a water temperature of 25 ± 1°C. Then, the mice were forced to swim for 6 min, and the video acquisition system was utilized to record. Use a stopwatch to observe the immobility time of the mice within 4 min after the experiment. The mice appear immobile, floating and losing struggle or just to ensure that the head is susceptible to the surface of the water that was regarded as immobile. After the test is expected to be completed, each mouse is dried with a towel and returned to the cage.

### Immunofluorescence labeling of brain slices

C57BL/6 J mice were anesthetized with sodium pentobarbital (45 mg/kg, i.p.) and fixed on a foam board in the supine position. The brain of mice was extracted and placed in a 4% paraformaldehyde solution for post-fixation overnight at 4°C as previously described [27]. Then, the brain tissues were dehydrated with 20% and 30% sucrose solutions at 4°C until the specimens completely settle to the bottom and sliced into 4 µm sections by using LeicaCM1900 and stored in PBS at 4°C. The slices were rinsed with 0.01 M PBS for 3 times and blocked with blocking solution consists of 3% bovine serum albumin at room temperature for 1 h. For immunostaining, the slices were incubated with the primary antibody 0.348 mg/ml EPK1 (dilution: 1:500, Cat. No. ab32537, Abcam), 1.155 mg/ml EPK2 (dilution: 1:500, Cat. No. ab184699, Abcam), 0.819 mg/ml CREB (dilution: 1:500, Cat. No. ab32096, Abcam) and 0.281 mg/ml BNDF (dilution: 1:500, Cat. No. ab108319, Abcam) overnight at 4°C followed by rinsed three times with 0.01 M PBS. Subsequently, the slices were incubated with the 0.5 mg/ml Alexa Fluor-conjugated secondary antibody (dilution: 1:500, Cat. No. ab209959, Abcam) at room temperature for 1 h. Then, nucleus was stained with DAPI at room temperature for about 2 min and quickly rinsed three times with 0.01 M PBS. The images were made by using a fluorescence microscope.

### Nissl staining

The mice were sacrificed and after 24 h to the last stressor of CUMS procedures. Each whole brain was rapidly dissected from mice and flushed in ice-cold saline as previously described [28]. The right hippocampi was separated on an ice bath and the left cerebral hemisphere was embedded in glue, and 6 µm of serial sections were performed in the coronal plane. Nissl staining was used to observe the morphology of hippocampus CA3 by microscopy. Image-Pro Plus 6.0 software was used to calculate the nerve cells in hippocampus CA3.

### Quantitative reverse transcription-polymerase chain reaction (qRT-PCR)

TRIzol reagent (9109, Takara) was utilized to extract the total RNA from brain tissues. 2 μg RNA was used to synthesize cDNA using BestarTM qPCR RT Kit (2220, DBI) according to the manufacturer’s instructions. qRT-PCR was performed using ABI 7500 instrument (ABI7500, ABI, Foster City, CA, USA) in a 20 μL reaction volume including qPCR master Mix (2043, DBI), 10 μL; each primer (10 μM), 0.5 μL; cDNA template, 1 μL and ddH_2_O, 8 μL. Amplification processes were as follows: 95°C, 2 min; 94°C, 20 s and 58°C, 20 s and 72°C, 20 s for 40 cycles. The relative expression of the genes was normalized to GAPDH. Relative expression levels were calculated by 2^−ΔΔCt^ methods as previously described [29]. The primers were listed as follow: GAPDH (forward: 5’-AGCCACATCGCTCAGACACC-3’; reverse: 5’-GTACTCAGCGCCAGCATCG-3’), ERK1 (forward: 5’-CCAAAGCAGAAAGGGTCGT-3’; reverse: 5’-ACACTCGGACCACCTCCTTC-3’), ERK2 (forward: 5’-CTTTACCGCTACGACGTGA-3’; reverse: 5’-GAAAGCACCCCTCCCATAG-3’), cAMP-response element binding protein (CREB) (forward: 5’-CCTTTGACTGTGATTTGTCC-3’, reverse: 5’-CAGTTACCTTGTCTTCCACT-3’) and CREB (forward: 5’-AACGGTCATTACATTCCGAC-3’, reverse: 5’-CACAATGGCAGCATTCCC-3’).

### Western blotting

Total protein from brain tissues was extracted by using RIPA Lysis Buffer (P0013, Beyotime, Shanghai, China) with 1 mM PMSF following the manufacturer’s instructions. 30 g proteins were boiled at 100°C for each sample with protein loading buffer for 5 min, followed by separation in 10–12% SDS-PAGE electrophoresis and transferred onto PVDF membranes. Then, 5% lipid-free milk/TBST buffer were used to block the membranes at room temperature overnight, incubated with anti-p-ERK1 + anti-ERK2 (ab76299, 1:5000, Abcam), anti-CREB (ab32515, 1:1000, Abcam), anti-BNDF (ab108319, 1:1000, Abcam) and anti-GAPDH (ab8245, 1:5000, Abcam) primary antibodies for 2 h at 4°C overnight, respectively. After being incubation with secondary antibodies anti-mouse IgG (BA1051, 1:20,000, BOSTR) or anti-rabbit IgG (BA1054, 1:20,000, BOSTR,) for 1–2 h at room temperature, the immuno-complexes were finally detected by ECL after washing by TBST and analyzed using the Image-Pro Plus 6.0 software [30].

### Statistical analysis

Data are shown as the mean ± standard deviation of at least three independent experiments. Statistical comparisons were performed using the unpaired *t* test between two groups, and one-way analysis of variance (ANOVA) with Tukey’s post hoc test was used to perform comparation more than two groups. Variables were tested at different time points using Bonferroni-corrected repeated measures ANOVA. All statistical analyses were completed with SPSS 21.0 software (IBM, Armonk, NY, USA), with two-tailed *p* < 0.05 as a level of statistical significance [31].

## Results

### CRS-induced depression-like behavior in mice

**Figure 1.**
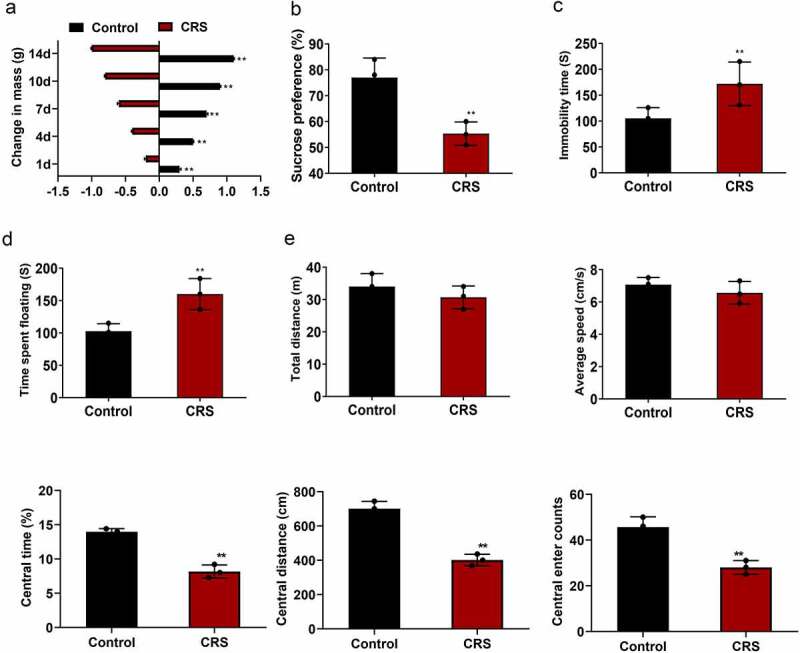
**CRS-induced depression-like behavior in mice**. (a) The weight loss of the mice during the CRS for 14 days. (b–d) SPT, TST and FST analysis of the mice in CRS group and control group, respectively. (e) Total distance, the average speed, the central area, central distance and the central enter times between CRS and control group detected by OFT. CRS, chronic restraint stress. Statistical comparisons were performed using the unpaired *t* test between two groups, *n* = 8. All data are presented as the mean ± SD; **p < 0.01, CRS group vs control group

In the present study, we aim to explore the effects of paeoniflorin on the CRS induced depression-like behavior in mice model and investigate the molecular mechanism of it. SPT, TST, OFT and FST were used to assess the depression symptoms. Immunofluorescence, qRT-PCR and western blotting were implemented to verify the ERK1/2 signaling pathway. First of all, CRS was utilized to induce depression-like behavior in mice. The depression-like behavior in mice was verified at 14 days after CRS. As shown in Figure 1a (mean ± SD, *p* < 0.01), the mice experienced CRS displayed a more prominent weight loss compared with the mice in normal group. Sucrose preference was dramatically reduced in the CRS group compared with the control group detected by SPT (Figure 1b, mean ± SD, *p* < 0.01). TST showed that the immobility time of mice was significantly increased in the CRS group compared with the control group (Figure 1c, mean ± SD, *p* < 0.01). FST also confirmed the results (Figure 1d, mean ± SD, *p* < 0.01). In addition, OFT results revealed that no difference was found in the total distance and the average speed between CRS and control group. However, the central area, central distance and the central enter time were significantly decreased in the CRS group compared with the control group (Figure 1e, mean ± SD, *p* < 0.01). These results showed that depression-like behavior was successfully induced by CRS.

### Paeoniflorin attenuated CRS-induced depression-like behavior

**Figure 2.**
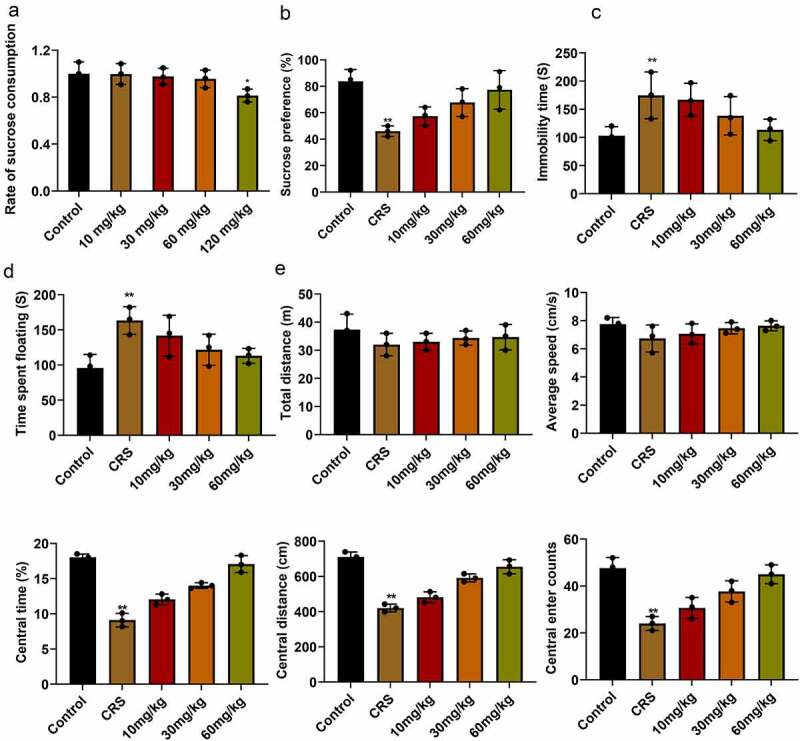
**Paeoniflorin attenuated CRS-induced depression-like behavior**. (a) Effects of paeoniflorin on the sucrose preference index in mice. (b) Sucrose preference was detected by SPT in CRS group treated with paeoniflorin (10, 30 and 60 mg/kg). (c and d) TST and FST to analyze the immobility time of mice in CRS group treated with paeoniflorin (10, 30 and 60 mg/kg). (e) Total distance, the average speed, the central area, central distance and the central enter times between CRS and control group detected by OFT. CRS, chronic restraint stress. Statistical comparisons were performed using one-way ANOVA to perform comparation more than two groups, *n* = 8. All data are presented as the mean ± SD; **p < 0.01, *p < 0.05, CRS group vs control group

At the beginning of the experiment, we first evaluated the sucrose consumption of mice at different concentrations. The results showed that when the concentration of paeoniflorin was less than 60 mg/kg, there is no difference between control group, while 120 mg/kg paeoniflorin markedly decreased sucrose consumption in mice (Figure 2a, mean ± SD, *p* < 0.05). In order to know the effect of paeoniflorin on CRS-induced depression-like behavior, the CRS mice were treated with paeoniflorin (10, 30 and 60 mg/kg). Sucrose preference was dramatically reduced in the CRS group compared with the control group detected by SPT (Figure 2b, mean ± SD, *p* < 0.01). TST showed that the immobility time of mice was significantly increased in the CRS group compare with the control group (Figure 2c, mean ± SD, *p* < 0.01). FST also confirmed the results (Figure 2d, mean ± SD, *p* < 0.01). In addition, OFT results revealed that no difference was observed in the total distance and the average speed between CRS and control group. However, the central area, central distance and the central enter time were significantly decreased in CRS group compared with control group (Figure 2e, mean ± SD, *p* < 0.01). In addition, we found that the degree of reduction in depressive-like behavior was elevated with the increase in dosage of paeoniflorin compared with the CRS group. And 60 mg/kg paeoniflorin was used for further study.

### Paeoniflorin attenuated the undesirable morphology and elevated the number of hippocampal neurons in CRS mice

**Figure 3.**
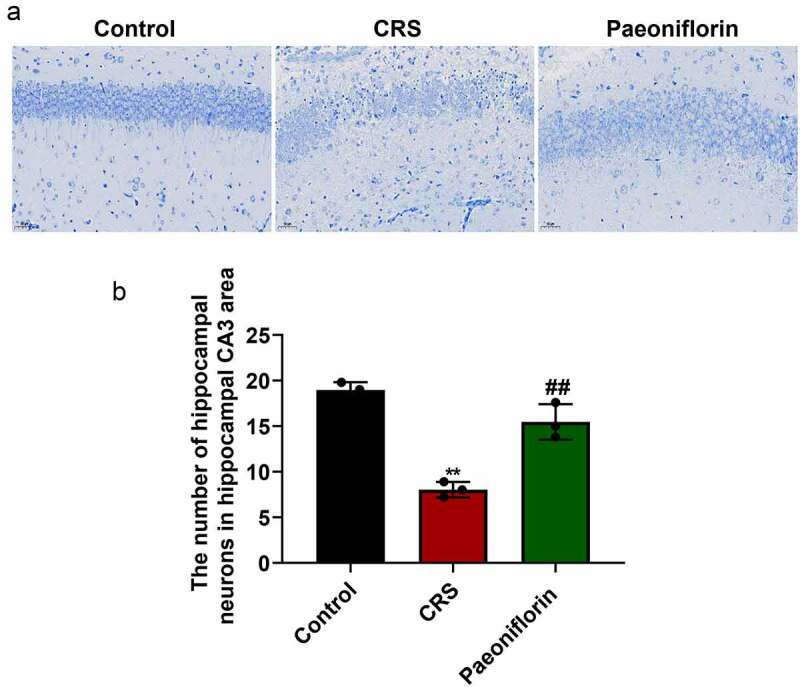
**Paeoniflorin attenuated the undesirable morphology and elevated the number of hippocampal neurons in CRS mice**. (a) The morphology of hippocampal neurons in hippocampal CA3 area of CRS-induced mice treated with 60 mg/kg paeoniflorin by Nissl staining, Bar = 100 μm. (b) The number of hippocampal neurons in hippocampal CA3 area of CRS-induced mice treated with 60 mg/kg paeoniflorin calculated by Image J. CRS, chronic restraint stress (*F* = 53.75, *n* = 8). Statistical comparisons were performed using the unpaired *t* test between two groups. All data are presented as the mean ± SD; **p < 0.01, CRS group vs control group. ^##^*p* < 0.01, Paeoniflorin group vs CRS group

In order to further confirm the role of paeoniflorin (60 mg/kg) on CRS-induced depression-like behavior, the morphology and number of hippocampal neurons in CRS-induced mice were investigated by Nissl staining. As shown in Figure 3a, the hippocampal neurons were disordered and loose with partial nucleus pyknosis, and shallow-dyed and partly dissolved Nissl substance was observed in the CRS group. Further analysis showed that the number of hippocampal neurons in hippocampal CA3 area was significantly reduced in the CRS group compared with control group (Figure 3b). In addition, the paeoniflorin could partially reverse the morphology and elevate the number of hippocampal neurons in CRS mice (Figures 3a and 3b, mean ± SD, *p* < 0.01). Taken together, these results indicated that paeoniflorin could attenuate CRS-induced depression-like behavior.

### Paeoniflorin attenuated CRS-induced depression-like behavior involved in ERK signaling pathway

**Figure 4.**
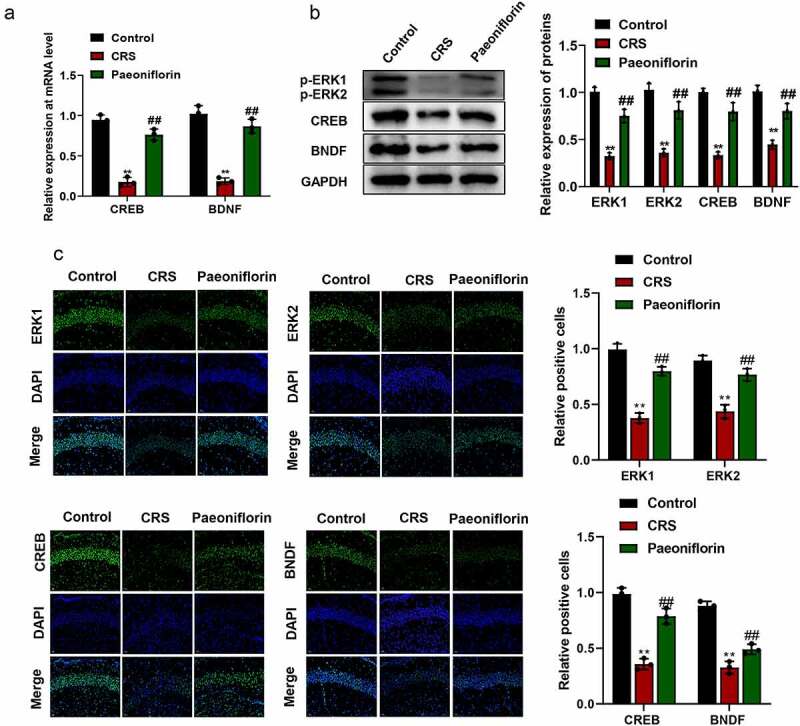
**Paeoniflorin attenuated CRS-induced depression-like behavior involved in ERK signaling pathway**. (a) The expression of CREB and BDNF detected by qRT-PCR at mRNA level in CRS mice treatment with 60 mg/kg paeoniflorin; GAPDH act as control (*F* = 129.1; *n* = 3). (b) The expression of p-ERK1, p-ERK2, CREB and BDNF detected by western blot at protein level in CRS mice treatment with 60 mg/kg paeoniflorin, GAPDH act as control (*F* = 323.8; *n* = 3). (c) Immunofluorescence staining was used to detect the expression of p-ERK1, p-ERK2, CREB and BDNF in CRS mice treatment with 60 mg/kg paeoniflorin, DAPI was used to stain the nuclei, Bar = 100 μm. CRS, chronic restraint stress (*F* = 62.73/10.47; *n* = 3). Statistical comparisons were performed using one-way ANOVA to perform comparation more than two groups. All data are presented as the mean ± SD; **p < 0.01, CRS group vs control group. ^##^*p* < 0.01, Paeoniflorin group vs CRS group

In order to know whether paeoniflorin affected depression-like behavior by affecting ERK signaling pathway, the expression of ERK1 and ERK2 and their downstream signaling molecules CREB and brain-derived neurotrophic factor (BDNF) were detected by qRT-PCR, western blot and immunofluorescence staining. The qRT-PCR and western blot assays results showed that the expression of p-ERK1, p-ERK2, CREB and BDNF were significantly downregulated in CRS group compared with control group while paeoniflorin could elevate the expression of these genes compared with CRS group (Figure 4a-b, mean ± SD, *p* < 0.01). In addition, immunofluorescence staining showed that the green fluorescent signal was declined in CRS group compared with control group while paeoniflorin could enhance the expression of these proteins compared with CRS group (Figure 4c, mean ± SD, *p* < 0.01). These results demonstrated that paeoniflorin attenuated CRS-induced depression-like behavior involved in ERK signaling pathway.

### ERK1/2 signaling inhibitor U0126 aggravated depression-like behavior

**Figure 5.**
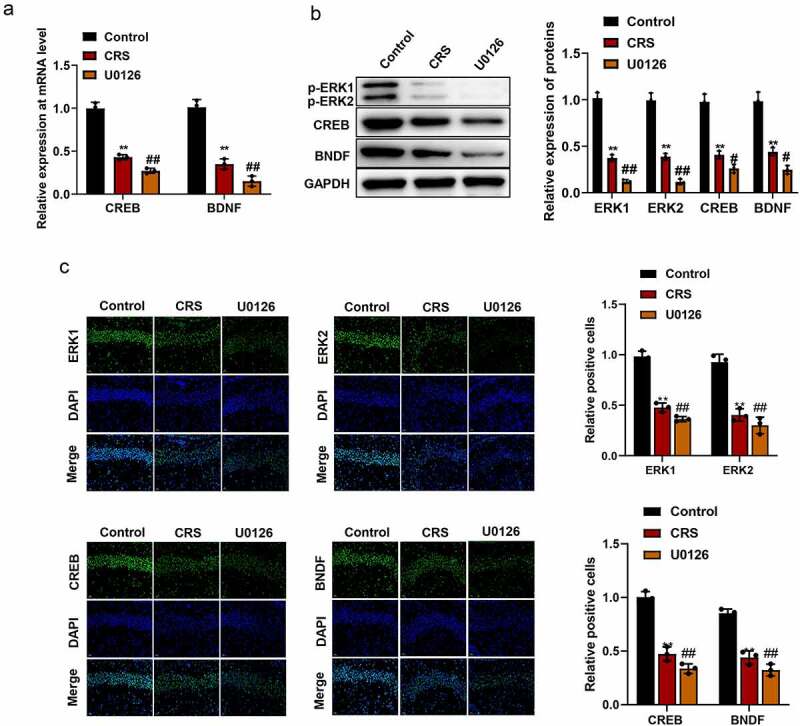
**ERK1/2 signaling-related genes was inhibited by ERK1/2 inhibitor U0126**. (a) The expression of CREB and BDNF detected by qRT-PCR at mRNA level treated by ERK1/2 inhibitor U0126; GAPDH act as control (*F* = 98.96; *n* = 3). (b) The expression of p-ERK1, p-ERK2, CREB and BDNF detected by western blot at protein level treated by ERK1/2 inhibitor U0126, GAPDH act as control (*F* = 288.5; *n* = 3). (c) Immunofluorescence staining was used to detect the expression of p-ERK1, p-ERK2, CREB and BDNF treated by ERK1/2 inhibitor U0126, DAPI was used to stain the nuclei, Bar = 100 μm. CRS, chronic restraint stress (*F* = 105.6/50.15; *n* = 3). Statistical comparisons were performed using one-way ANOVA to perform comparation more than two groups. All data are presented as the mean ± SD; **p < 0.01, CRS group vs control group. ^##^*p* < 0.01, ^#^*p* < 0.05, U0126 group vs CRS group

**Figure 6.**
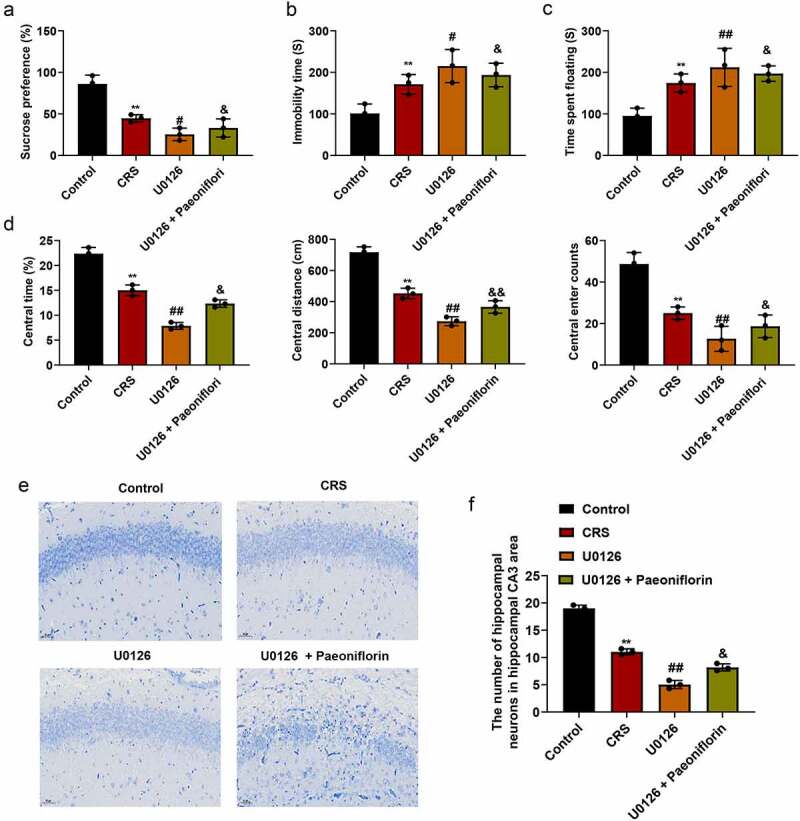
**ERK1/2 signaling inhibitor U0126 aggravated depression-like behavior**. (a) Sucrose preference detected by SPT when treated with U0126 (*F* = 28.4; *n* = 8). (b-c) The immobility time of mice detected by SFT and TST in different groups, respectively (*F* = 8.473/9.985/115.7/91.24/28.15; *n* = 8). (d) Total distance, the average speed, the central area, central distance and the central enter times between CRS and control group detected by OFT. (e) The morphology of hippocampal neurons in hippocampal CA3 area of CRS-induced mice treated with U0126 by Nissl staining, Bar = 100 μm. (f) The number of hippocampal neurons in hippocampal CA3 area of CRS-induced mice treated with U0126 calculated by Image J. CRS, chronic restraint stress (*F* = 257.4; *n* = 8). Statistical comparisons were performed using one-way ANOVA to perform comparation more than two groups. All data are presented as the mean ± SD; **p < 0.01, CRS group vs control group. ^##^*p* < 0.01, ^#^*p* < 0.05, U0126 group vs CRS group; ^&&^*p* < 0.01, ^&^*p* < 0.05, U0126 and paeoniflorin group vs U0126 group

In order to further confirm the effect of ERK signaling pathway in the depression-like behavior, mice were treated with ERK1/2 signaling pathway specific inhibitor U0126. As shown in Figures 5a and 5b (mean ± SD, *p* < 0.05/0.01), the expression of p-ERK1, p-ERK2, CREB and BDNF were significantly downregulated in CRS group, and when treated with U0126, the expression of p-ERK1, p-ERK2, CREB and BDNF was further decreased compared with CRS group at mRNA and protein levels Immunofluorescence staining (Figure 5c, mean ± SD, *p*< 0.01) was also confirmed the results at protein level. Further analysis showed that the sucrose preference was dramatically reduced (Figure 6a, mean ± SD, *p* < 0.05/0.01) and the immobility time of mice was significantly increased (Figures 6b and 6c, mean ± SD, *p* < 0.05/0.01) in U0126 group compare with CRS group. In addition, OFT results revealed that the central area, central distance and the central enter time were significantly decreased in U0126 group compared with CRS group (Figure 6d, mean ± SD, *p* < 0.05/0.01). Furthermore, we found that the disorder and loose of hippocampal neurons, nucleus pyknosis and cell damage was exacerbated when treatment with U0126 (Figure 6e). And the number of hippocampal neurons in hippocampal CA3 area was significantly reduced in the U0126 group compared with CRS group (Figure 6f, mean ± SD, *p* < 0.05/0.01). In addition, we found that paeoniflorin could partially reverse the depression-like behavior and the injury of hippocampal neurons caused by U0126 (Figure 6a to 6f).

## Discussion

Depression is the most common serious psychiatric disorder among those with established sets of emotional and cognitive symptoms [32,33]. Several antidepressant drugs have used for the treatment of depression, such as serotonin–noradrenaline reuptake inhibitors, SSRIs and tricyclic antidepressants [34]. However, these drugs did not show a good effect and confer acute adverse effects. Therefore, it is urgent to develop new antidepressants for depression.

Paeoniflorin is a monoterpene glycoside compound, which was first extracted from the Paeonia lactiflora in 1963 [35]. It is the main active component of the glycoside substance in the root of Paeonia lactiflora [36,37]. In recent years, growing evidences have shown that paeoniflorin has an important medicinal value for its antitumor, anti-oxidant, immune-regulating properties and has less toxic and side effects [38,39]. In addition, increasing number of studies also proved that paeoniflorin has an antidepressant effect by reducing neuroinflammation in the brain area [27,40,41]. For instance, pretreatment with 20 or 40 mg/kg paeoniflorin for 4 weeks can reverse the depression-like behavior and abnormal inflammatory cytokine levels in serum, medial prefrontal cortex, ventral hippocampus and amygdala [27]. Recently, it showed that paeoniflorin attenuated impairment of spatial learning and hippocampal long-term potentiation in mice subjected to CUMS [19]. And Hu et al. also demonstrated that paeoniflorin could ameliorated the symptoms and improve the functional capability of post-stroke depression (PSD) rats, similar to the effect of fluoxetine [42,43]. In consistent with these findings, in the present study, we aimed to confirmed that paeoniflorin could ameliorate chronic stress-induced depression-like behavior in mice and explore its novel molecular mechanism of action. These results would provide more support for the antidepressant effect of paeoniflorin.

Increasing evidence supports a pivotal role of the MAPK, in particular the ERK subclass of MAPKs [44]. In humans and various chronic animal models of depression, the ERK signaling was significantly downregulated in the prefrontal cortex and hippocampus, two core areas implicated in depression [45]. Among several ERK isoforms (ERK1/2/3/4/5/7), ERK1/2 have been most thoroughly investigated and characterized in the central nervous system [46]. It has been demonstrated that paeoniflorin could participate in various kinds of diseases by regulating signaling pathways. Such as paeoniflorin inhibits IL-1beta-induced MMP secretion via the NF-κB pathway in chondrocytes in the pathogenesis of osteoarthritis (OA) [47]. And it improved pressure overload-induced cardiac remodeling by modulating the MAPK signaling pathway in spontaneously hypertensive rats [48,49]. The ERK cascade is an important signaling pathway in the cells, which involved the initiation and regulation of various stimulus information outside the cell. For example, Yuan et al. showed that inosine increased the activity of ERK and CREB in the hippocampus and prefrontal cortex. It alleviated depression-like behaviors in adolescent rats via regulating ERK-CREB signal system [50]. Li et al. revealed that acupuncture intervention has an antidepressive role in CUMS-induced depression rats pertained to its effects in up-regulating the expression of p-ERK1/2 and BDNF in the prefrontal cortex tissue [51]. Otherwise, brain permeability is one of the factors affect depression, studies have shown that the increased ERK expression would reduce brain permeability to ameliorate depression [52]. However, the ERK signaling pathway affected by paeoniflorin in depression is less studied. In the present study, we demonstrated that the expression of key proteins involved in ERK signaling pathway was dramatically downregulated when experienced chronic restraint stress, including ERK, CREB and BDNF. However, paeoniflorin could attenuate the reduction of the expression for ERK, CREB and BDNF. In order to verify this signal pathway, specific ERK inhibitor U0126 was used to block the pathway, and observed the spontaneous activities and the change of the key molecular effects of the signaling pathway in CRS model group. The results revealed that the expression of ERK, CREB and BDNF was also sharply decreased when treated with ERK inhibitor U0126. However, this study still has some limitations. For example, we did not provide evidence to indicate other signaling pathways were not involved in the antidepressive effect conferred by paeoniflorin that has multiple targets. In the next step, we will explore multiple targets to make its molecular mechanism is more abundant.

## Conclusion

In summary, we demonstrated that paeoniflorin treatment decreased the degree of depression in the CRS mice. And further analysis showed that paeoniflorin attenuated chronic stress-induced depression-like behavior in mice by affecting the ERK1/2 pathway. These findings provided the basis for the molecular mechanism of paeoniflorin on the effect of depression, which support paeoniflorin might act as an important drug in the treatment of depression.

## Data Availability

The datasets used and/or analyzed during the current study are available from the corresponding author on reasonable request.

## Highlights

1. This study explored the effect and the molecular mechanism of paeoniflorin on depression in a CRS mice model.

2. The study demonstrated that paeoniflorin attenuated chronic stress-induced depression-like behavior in mice by affecting ERK1/2 pathway.

3. These findings provided basis for the molecular mechanism of paeoniflorin on the effect of depression, which support paeoniflorin might act as an important drug to treatment of depression.
